# Oldoini in the Treatment of Croup

**Published:** 1877-11

**Authors:** 


Oldoini on the Treatment of Croup.—Dr. S. Oldoini relates in the *Annati, Universali* for March, five cases of croup observed during the epidemic at Spezzia, in which he successfully employed copaiba and cubebs. His plan was to give to adults, every two hours, a dessert-spoonful of a syrup composed of 14 grammes (about 5 ounces) of balsam of copaiba, 20 grammes (about 7 ounces) of powdered gum, 50 grammes (about 17½ ounces) of water, and 14 drops of essence of mint; and also, every two hours, a table-spoonful of a mixture consisting of 12 grammes (186 grains) of recently powdered cubebs, and 24 grammes (8 ounces) of syrup. For children, the dose was reduced. The malady disappeared in a period of two or three days, rarely extending to seven.

Four of the five cases were children under four years of age; some affected with simple croup, others with croup complicated with diphtheria. The condition of the patients, when first put under treatment, was very grave; there was high fever, the submaxillary glands were engorged, the voice and crying were weak, the cough harsh, and there was marked dyspnœa. The beneficial effects of the medicine above described occurred without the use of emetics, mercurials, or any other treatment.

